# Severity of Bone Marrow Edema on MRI Predicts the Diagnostic Potential of Foot Joint Injections

**DOI:** 10.1177/10711007251334097

**Published:** 2025-05-05

**Authors:** Matthäus Cieciera, Reto Sutter, Stephan H. Wirth, Tobias Götschi, Nadja A. Farshad-Amacker

**Affiliations:** 1 Department of Radiology, Balgrist University Hospital, University of Zurich, Zurich, Switzerland; 2 Department of Orthopedics, Balgrist University Hospital, University of Zurich, Zurich, Switzerland; 3 Unit for Clinical and Applied Research, Balgrist University Hospital, University of Zurich, Zurich, Switzerland

**Keywords:** foot, pain, MRI, injections, intra-articular, diagnostic imaging

## Abstract

**Background:**

Intraarticular steroid and local anesthetic injections are often performed for foot pain. Localizing the exact joint acting as a pain generator in foot pain is eminent for optimal and cost-effective treatment. Because of the complex anatomy of the foot with multiple small articulations side by side, this can be challenging. This study’s purpose was to determine magnetic resonance imaging (MRI) findings as possible predictive factors for the immediate outcome of intraarticular injections of the foot.

**Methods:**

All single joint foot injections at our institution from January 2019 to May 2020 with prior MRI scans were included in this retrospective study. Visual analog scale (VAS) pain assessments before and after injection, relative change in pain scores and indication were recorded. All MRIs were retrospectively analyzed by 2 blinded radiologists regarding the presence and severity of subchondral bone marrow edema (BME), subchondral cysts, cartilage defects, osteophytes, joint effusion, and soft tissue edema. Spearman analysis was used to assess correlation between MRI findings and pain relief. Interrater variability was assessed using weighted Cohen κ analysis.

**Results:**

A total of 164 injections from 162 patients were included (mean age, 53 years ± 15.5, 99 females). Relative pain reduction correlated significantly with BME severity (*P* < .05). Interrater reliability assessing BME was excellent (weighted Cohen κ 0.863).

**Conclusion:**

The degree of pain reduction after intraarticular foot injections correlates significantly with the severity of subchondral bone marrow edema–like signal on MRI before injection.

## Graphical abstract

**Graphical Abstract:**
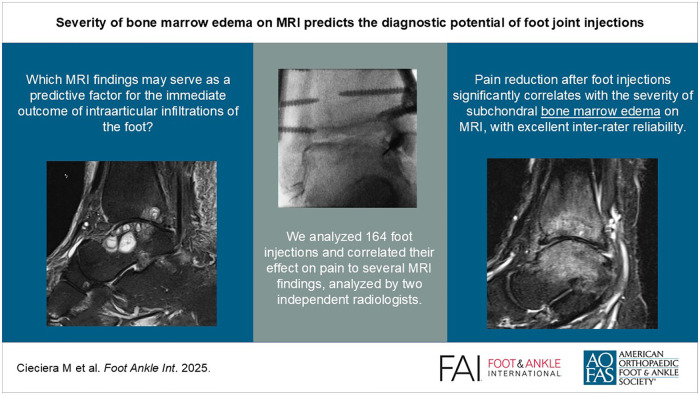
This is a visual representation of the abstract.

**Level of Evidence:** Level III, retrospective cohort study.

## Introduction

Foot pain is a very common medical condition in the aging population worldwide, with a prevalence of up to 63% of people reporting foot pain, aching, or stiffness in primary care consultations for musculoskeletal foot and ankle problems.^21^ With efforts in keeping health care cost low, cost-effective treatments are desired. Because of the complex anatomy of the foot with multiple small articulations side by side, localizing the exact source of pain can be difficult during clinical examination, with imaging giving an aiding perspective regarding a wide variety of underlying pathologies.^20,24^ To localize the exact joint acting as a pain generator is getting increasingly imminent for optimal and cost-effective treatment, as multiple simultaneous joint injections are nowadays avoided.

Fluoroscopy-guided intraarticular injections with local anesthetics are an effective and safe way to confirm the source of pain, often achieving immediate pain relief. Further, as it is usually combined with concomitant administration of corticosteroids, longer-lasting treatment of pain can often be achieved simultaneously, acting as an efficient, safe, and cost-effective treatment to stave off or lengthen duration until surgical treatment is needed.^13^

In some patients, however, injection does not yield in satisfactory pain relief^26^ and in some cases several intraarticular injections into different joints are needed to localize the exact source of pain.^16,19,22^

Magnetic resonance imaging (MRI) is a commonly used and widely available modality for assessing the structures of the foot, including the bones, cartilage, ligaments, and tendons, in order to identify underlying pathology.

Bone marrow edema (BME) on MRI refers to areas of high signal intensity within bone marrow, seen on highly fluid-sensitive MRI sequences. It reflects a variety of underlying histologic changes, including inflammation, necrotic or remodeled bone trabeculae, fibrosis, hemorrhage, as well as edema. As such, BME is a broad and nonspecific term with various potential causes, for example, in fractures, infections, inflammations, tumors, or reactive edema, which may occur in response to cartilage defects or even stress-induced changes.^1–5,8–12,16,17,23,27,31,32^

To our knowledge, evidence regarding feasibility and effectiveness of intraarticular injections of the foot is scarce, and MRI findings such as presence and extent of BME, among others, have not yet been correlated with degree of pain relief immediately after intraarticular injections of the foot and have not been used as a predicting tool to localize pain source before an injection. Our hypothesis was that specific MRI findings could predict the response to diagnostic injections in foot joints. Therefore, the aim of this study was to identify potential predictive parameters on previous MR images that could help forecast the effectiveness of pain reduction following an intraarticular foot joint injection, thereby guiding clinical decision making on which joint to inject first.

## Methods

The study was approved by the local ethics committee (BASEC Nr 2020-02469). Informed consent was obtained from all participants regarding the injection and use of their acquired data for research.

### Patients

All patients aged ≥18 years who received a fluoroscopy-guided intraarticular joint injection of the foot at our institution from January 1, 2019, to May 31, 2020, and had an MRI of the foot performed no more than 1 month before the injection were included in this retrospective study. Indications for the MRIs were “foot pain,” as referred by our foot specialists of the University Foot Center.

Exclusion criteria were injections of more than 1 articulation of the foot, except for more than 1 Lisfranc articulation, as there often is communication between the compartments of several Lisfranc articulations; injection of an extraarticular site, such as injection of the plantar fascia; poor MRI image quality or incomplete visualization of the respective articulation; arthroplasty; direct injection of a coalition; or incomplete pain assessment. One patient had an injection of the space between talar, navicular, calcaneal, and cuboidal bone and was excluded because of this uncommon injection.

Presence of any previous foot surgery and indication for injection was noted for each patient. Patients with “bone marrow replacements” such as in osteomyelitis or in neoplastic disease were not included in this study.

### MRI

MRIs were performed on one of 4 different MRI scanners in our institution: two 1.5-tesla (T) (Siemens Magnetom Avanto-fit) and two 3-T (Siemens Vida). To be included in our study, each patient's MRI had to consist of at least 1 fat-saturated fluid-sensitive and 1 non-fat-saturated PD sequence fully depicting the articulation in question.

### Pain Assessment

At our institution, it has been well established for over 15 years to give every patient an explanation of the VAS pain score system by our technicians. Patients are then asked to complete a pain assessment before and 15 minutes after the injection, using a visual analog scale board ranging from 0 (no pain) to 10 (excruciating pain). Patients with pain limited to certain movements or activities were asked to reproduce the painful movement or position after injection to properly assess pain relief. Pain assessments were stored digitally and retrieved for each patient.

### Joint Injection

The intraarticular injection was performed under fluoroscopic guidance by a trained radiologist using a 25-gauge needle, using an anterior approach for ankle joints, a posterolateral approach for subtalar joints, a lateral approach for calcaneocuboidal joints, and dorsal approaches for talonavicular, naviculocuneiform, intercuneiform, Lisfranc, metatarsophalangeal, and interphalangeal joints.^7,14^ After needle placement, 0.3-1 mL of contrast material (Iopamiro 200, iopamidol, 200 mg iodine per milliliter; Bracco Suisse SA) was injected. A fluoroscopic radiograph was obtained to confirm intraarticular access. Then, a maximum of 1 mL of crystalloid corticosteroid suspension (Triamcort, Triamconolon 40 mg/mL; Helvepharm AG) and a maximum of 5 mL of local anesthetic (0.5-5 mL depending on the size of injected joint, Rapidocain 2%, lidocaine 20 mg/mL; Sintetica SA) was injected.

### MRI Analysis

Image analysis was conducted by a junior radiologist (M.C.) with 1 year of experience and a senior radiologist (N.F.), with >9 years of experience. Both are MSK-fellowship-trained and word at a specialized musculoskeletal (MSK) radiology department in a dedicated orthopaedic university hospital. Both readers were blinded to each other, to the pain assessments before and after treatment and to the clinical indications for injections.

**Figure 1.**
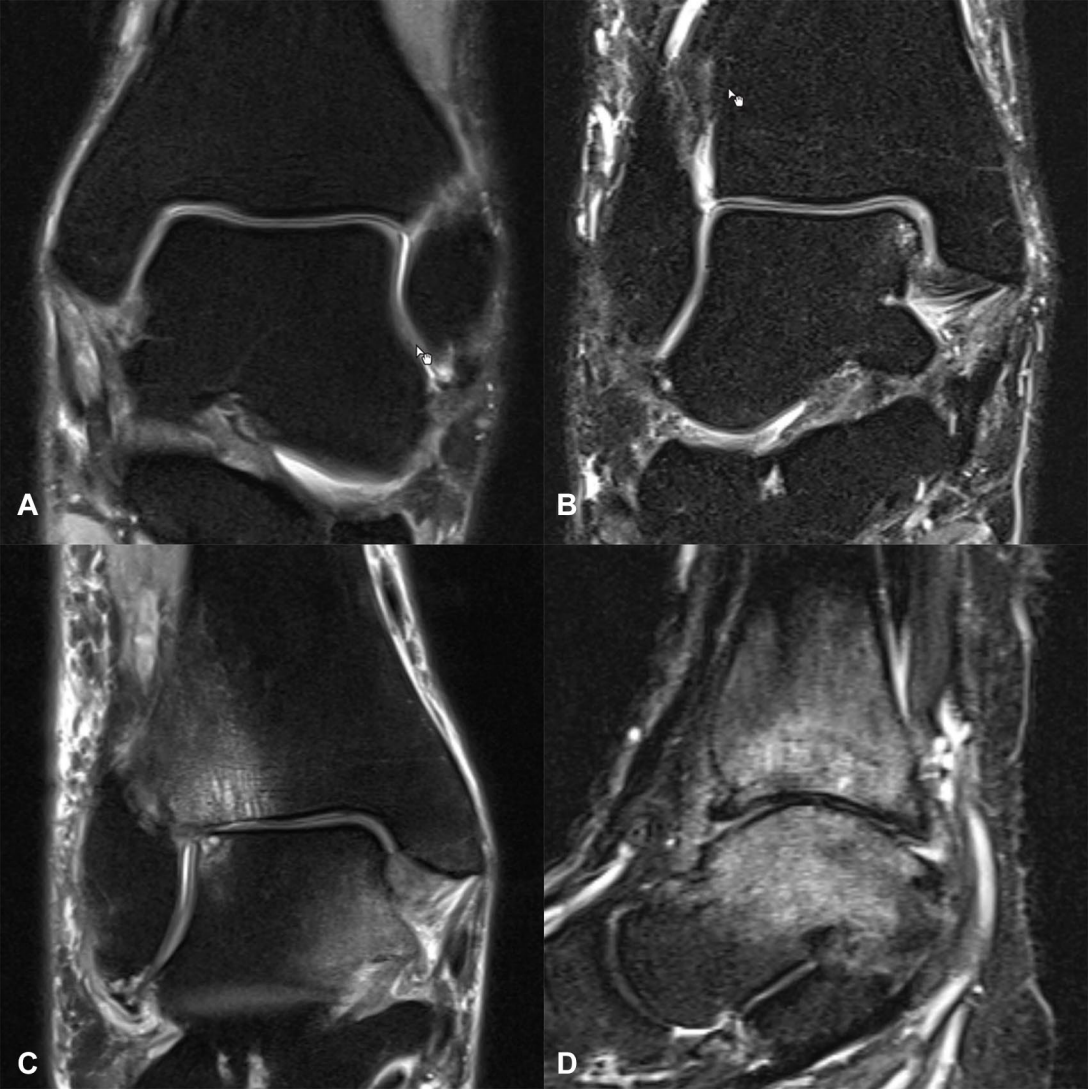
Grading of the presence and extent of BME-like signal on fluid-sensitive magnetic resonance images of 4 different patients: (A) none; (B) mild BME with slight involvement of only 1 articular side, no involvement more than 1 cm below cartilage surface; (C) moderate BME with both joint sides involved, involvement also >1 cm below the cartilage surface; (D) severe BME involving both sides of the cartilage surface. BME, bone marrow edema.

**Figure 2.**
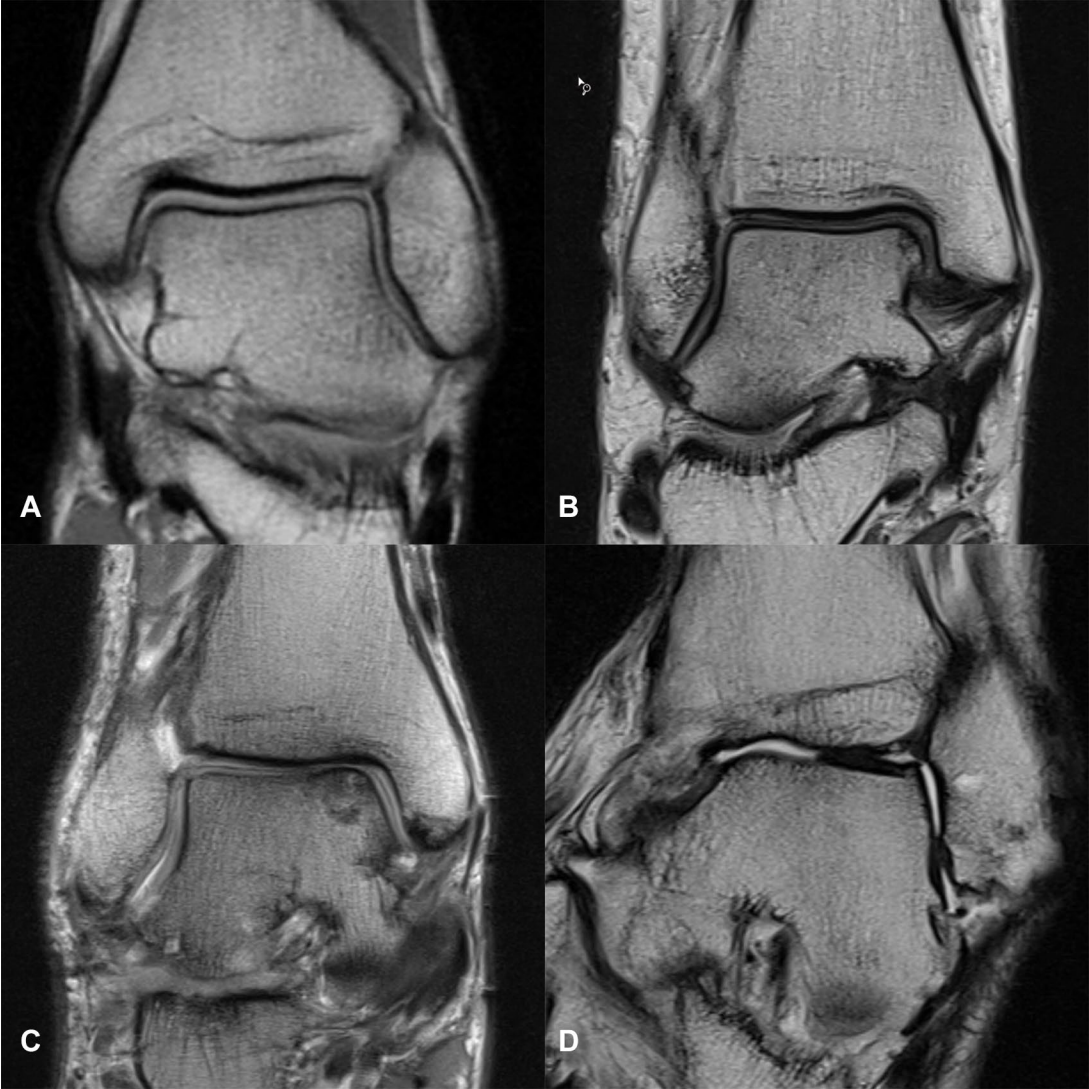
Grading of deep cartilage damage on proton density–weighted magnetic resonance images of 4 different patients: (A) none, (B) mild, (C) moderate, and (D) severe.

The dimension of subarticular BME, as well as the extent of the BME in distance to the articular cartilage (greater and less than 1 cm), subchondral cyst formation, cartilage defects, osteophyte formation, joint effusion, and soft tissue edema were documented. Presence and severity of each mentioned variable was analyzed grading from 0, none; 1, mild; 2, moderate; to 3, severe (Figures 1 and 2).

### Statistical Analysis

#### Pain assessments

The response to treatment in terms of pain was quantified by the relative change in pain score as assessed by a visual analog scale (ranging from 0 to 10) and calculated as: $\Delta \mathrm{VAS}=({\mathrm{VAS}}_{\mathrm{post}}-{\mathrm{VAS}}_{\mathrm{pre}})/{\mathrm{VAS}}_{\mathrm{pre}}$ . Any decrease in VAS was regarded as pain relief. Frequency of pain relief was described for all patients, and for each injected articulation separately.

#### Interrater reliability

For assessment of interrater reliability, MRI interpretations were compared using a weighted Cohen κ for each assessed parameter.

#### MRI-VAS pain score correlations

Spearman rank correlation test was used to assess correlation between MRI findings and VAS degree of pain before treatment and VAS degree of pain after treatment, with relative pain reduction for all MRI parameters.

Spearman analysis was again used to assess possible correlations between MRI parameters and relative pain reduction for ankle, subtalar, Lisfranc, and metatarsophalangeal joints. The other joints were not included in this separate analysis due to a too-small sample size in those joints.

Sensitivity, specificity, accuracy, positive predictive values, and negative predictive values in predicting a positive response to intraarticular injection (any decline in VAS) were calculated for each MRI finding (presence of finding, any grade 1-3).

A *P* value of below .05 was considered statistically significant. Interrater agreement was graded according to Landis and Koch.^18^ Author T.G. performed the statistical analyses.

## Results

**Figure 3.**
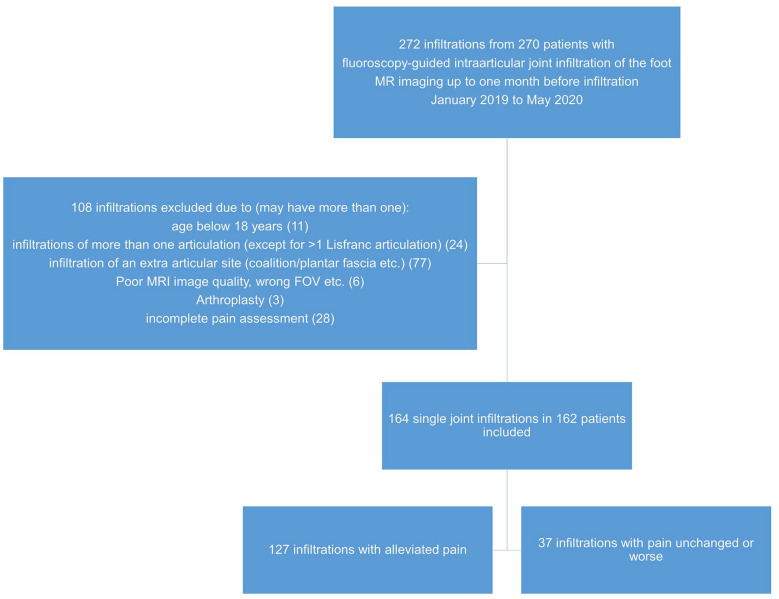
Flowchart of the study, from initial retrieval to final study cohort.

A total of 272 single joint injections were retrieved from the PACS search, of which 164 single joint injections in 162 patients (99 females, 63 males) were included in this retrospective study (Figure 3, Table 1).

**Table 1.** Patient Demographics.

Characteristic	Value
Age	
Min-Max	18-83
Mean ± SD	51.4 ± 15.5
Sex, n (%)	
Male	63 (39)
Female	99 (61)
Total	162(100)
Infiltrated articulation, n (%)	
Ankle	75 (46)
Subtalar	28 (17)
Metatarsophalangeal	28 (17)
Lisfranc	17 (10)
Calcaneocuboidal	6 (4)
Naviculocuneiform	4 (2)
Talonavicular	5 (3)
Interphalangeal	1 (1)
Total	164 (100)
Days from MRI to infiltration	
Min-max	0-29
Mean	8.4
Prior surgery, n (%)	
Yes	66 (40)
No	98 (60)
Indications for infiltration, n (%)	
Osteoarthritis	72 (44)
Impingement	43 (26)
Instability	10 (6)
Pain	30 (18)
Posttraumatic pain	5 (3)
Postsurgical pain	6 (4)
Pes planovalgus	10 (6)
Osteochondral lesion	10 (6)
Hallux valgus	6 (4)
Coalition	2 (1)
Os trigonum	1 (1)
Morbus Köhler	3 (2)

Abbreviation: MRI, magnetic resonance imaging.

One patient had an injection of 2 different articulations with 26 days between sessions, 1 patient had an injection of both left and right ankle joints, both patients documented each injection with a separate pain assessment. Mean patient age was 51.5 years (SD 15.5 years, range 18-83 years) at date of injection. Forty percent of patients had prior surgery, such as collateral ligament repair or arthroscopic ankle debridement. No patient had metallic implants relevantly affecting image quality. Mean time from MRI to injection was 8.4 days (SD 7.6 days, range 0-29 days). Most common indications were osteoarthritis (44%), impingement (26%), and pain not further classified (18%). The most common injected articulation was the ankle joint (n = 75 injections, 46% of all injections), followed by the subtalar joint (n = 28, 17%), one of the metatarsophalangeal joints (MTPJs) (n = 18 first MTPJ, n = 9 second MTPJ, n = 1 fourth MTPJ), and one of the Lisfranc joints (n = 17, 10%).

The mean pain score before injection was 5.38 (SD ± 2.36, range 0-10). The mean pain score after injection was 2.65 (SD ± 2.17, range 0-10), and the average relative pain reduction was −47% (SD ± 37%, range −100% to +67%). In 37 of 164 (23%) of all injections, no pain relief was achieved.

For reader 1 and reader 2, positivity rates for assessed MRI parameters for included patients were comparable (Table 2).

**Table 2.** Positivity Rates for All Assessed MRI Parameters for Both Interpretations.

	Positivity Rate, %	
Variable	Reader 1	Reader 2
BME	72.0	69.5
BME <1 cm below cartilage	71.3	68.3
BME >1 cm below cartilage	41.5	37.2
Subchondral cysts	33.5	43.3
Deep cartilage damage	73.2	58.5
Soft tissue edema	69.5	50.0
Osteophyte formation	53.7	59.8
Effusion	58.5	71.3

Abbreviations: BME, bone marrow edema; MRI, magnetic resonance imaging.

**Figure 4.**
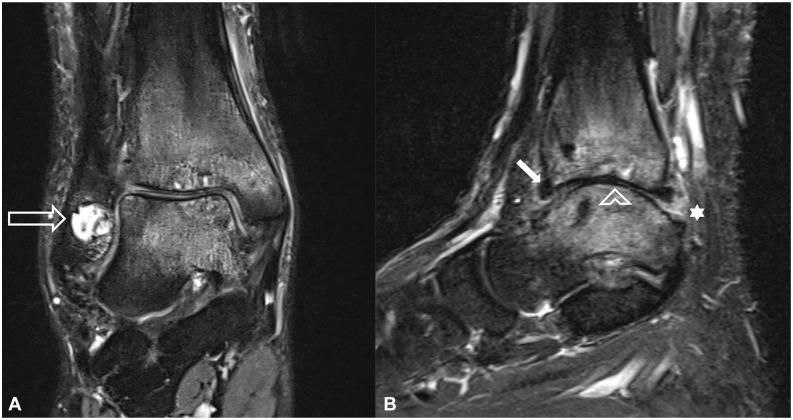
MRIs before fluoroscopy-guided intraarticular injection of an ankle joint in a 40-year-old female patient shown on (A) coronal PD Dixon fat suppressed and (B) sagittal STIR. MRIs show severe bone marrow edema, subchondral cyst formation (open arrow), moderate cartilage damage (arrowhead) and soft tissue edema (asterisk), and little osteophyte formation (arrow). Patient responded very well to treatment, with a decline in VAS score from 5 before to 0 after injection (100% decline). MRIs, magnetic resonance images; PD, proton density; STIR, short tau inversion recovery; VAS, visual analog scale.

**Figure 5.**
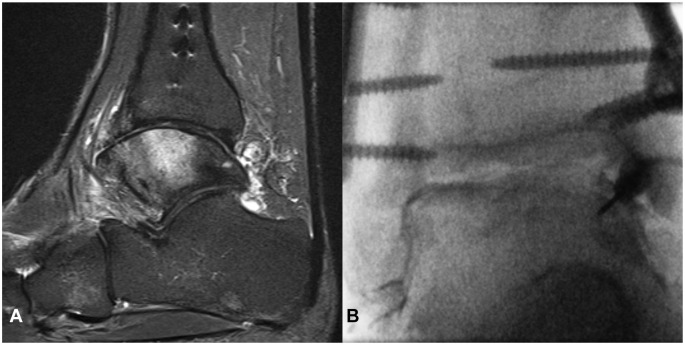
(A) Sagittal STIR magnetic resonance image before fluoroscopy-guided intraarticular injection of an ankle joint of a 29-year-old man with posttraumatic osteoarthritis shows severe bone marrow edema and severe cartilage damage in the ankle joint. (B) corresponding fluoroscopic image taken during injection. This patient responded very well to treatment, with a decline in VAS from 4 before to 0 after injection (100% decline). STIR, short tau inversion recovery; VAS, visual analog scale.

Interrater reliability results ranged from almost perfect, such as for the assessment of BME (0.863), to fair, when assessing soft tissue edema (0.342). Correlation values of one reader, the one with more clinical experience in musculoskeletal imaging (>9 years) is reported in further analysis (see Table 3 for interrater reliability results and correlations). Significant correlations were reported assessing BME and joint effusion in comparison with higher relative decrease in VAS pain scores (correlation coefficient −0.16 to −0.2; *P* < .05) (Figures 4 and 5).

**Table 3.** Interrater Reliability and Pain Change: Correlations.^a^

Variable	Cohen κ (95% CI)	Relative VAS Change	*P* Value
BME	0.863 (0.807-0.919)	−0.19	<.05
BME <1 cm below cartilage	0.834 (0.774-0.893)	−0.20	<.05
BME >1 cm below cartilage	0.593 (0.479-0.706)	−0.17	<.05
Subchondral cysts	0.600 (0.500-0.699)	−0.10	n.s.
Deep cartilage damage	0.597 (0.504-0.690)	−0.13	n.s.
Soft tissue edema	0.342 (0.231-0.452)	−0.12	n.s.
Osteophyte formation	0.482 (0.374-0.590)	−0.09	n.s.
Effusion	0.388 (0.2760-0.500)	−0.16	<.05

Abbreviations: BME, bone marrow edema–like signal; MRI, magnetic resonance imaging; n.s., not significant; VAS, visual analog scale.

a The table presents assessed MRI parameters with their respective interrater reliability using a weighted Cohen κ, plus correlations of each MRI parameter and relative changes in VAS pain scores, using Spearman rho correlation tests and data of the more experienced reader, with significance level of the correlations.

Subgroup analysis of each joint showed the same trends for the Lisfranc joints, the ankle joints, and the metatarsophalangeal joints, although not for the subtalar joint. Other joints, such as the talonavicular joint, were not separately analyzed because of too-small sample sizes.

Sensitivity, specificity, accuracy, positive and negative predictive value for each MRI parameter in predicting a positive response to intraarticular injection (with a cut-off of a relative decline of VAS ≥50%) are summarized in Table 4.

**Table 4.** Sensitivity, Specificity, Accuracy, PPV and NPV.

Variable	Sensitivity, %	Specificity, %	Accuracy	PPV, %	NPV, %
BME	72.0	33.0	0.54	54.0	52.0
BME <1 cm below cartilage	71.0	35.0	0.54	54.0	52.0
BME >1 cm below cartilage	41.0	67.0	0.53	57.0	50.0

Abbreviations: BME, bone marrow edema–like signal; PPV, positive predictive value; NPV, negative predictive value.

The postoperative state did not seem to substantially influence the efficacy of injection, as the nonresponder rate in patients without prior surgery was comparable to the nonresponder rate in patients with prior surgery (25.5% and 18.2%, respectively; difference not significant using a Student *t* test; total nonresponder rate = 22.6%).

## Discussion

To help clinical decision making toward single joint injection in the foot, our study’s purpose was to find possible predictive parameters on MRI predicting the diagnostic value of an intraarticular joint injection. We were able to demonstrate that the presence and extent of bone marrow edema–like signal significantly correlated with pain reduction after intraarticular injection, with excellent interrater reliability.

Correlations of pain relief with BME were significant in both interpretations. Cartilage damage (reader 2) and effusion (reader 1) showed significance in one of the readers only. Cartilage damage and joint effusion might be harder to objectively quantify, given the only fair interrater reliability, and therefore does not seem to be a good predicting parameter to be used. BME, subchondral cysts, and joint effusion as possible predictors for effectiveness of joint injections were reported by Strobel et al^26^ after acromioclavicular joint injection. In contrast to Strobel et al, where pain relief was significantly related to capsular hypertrophy, this parameter was not assessed in our study, as this descriptive term is rarely used to describe joints of the foot.

Other studies that sought to demonstrate the predictive value of MRI in the context of injection are rare, and those we are aware of reached mixed results. Hofmann et al^15^ did not find any relevant correlation of MRI parameters with reported pain after facet joint injection; however, BME was not among the MRI parameters examined in their study. In a meta-analysis comparing intraarticular injection analgesia for arthroscopic shoulder surgery with systemic analgesia or interscalene brachial plexus block, intraarticular injection analgesia appeared to be superior regarding pain control, opioid consumption, and patient satisfaction.^30^

For our study, we used a standardized and reproducible approach to grade parameters of joint degradation for all cartilaginous articulations of the foot. There are proposed grading systems for MRI assessment of osteoarthritis especially of the knee; however, there is no broadly accepted and used classification system for grading of osteoarthritis of the foot.^6^ The scoring system used for this study was inspired by existing scoring systems and commonly used descriptors used in daily clinical practice. Following clinical practice, a simple yes/no approach for MRI description was avoided.

We defined a cut-off for pain relief as a VAS decrease of ≥50% for assessment of sensitivity and specificity, in line with Spirig et al,^25,26^ as opposed to Strobel et al, who used a cut-off value of ≥70% for an adequate pain relief. We chose ≥50% because we believe this level of pain relief can be accurately evaluated by patients (pain reduced by more than half). In contrast, a further decrease to 70% may be difficult for patients to perceive and could introduce bias. For example, if initial pain is rated 4, it is easy for patients to recognize a reduction by half (down to 2). However, distinguishing between VAS score 2 from 1 (a 70% reduction as there is no decimal point) might be challenging for patients to evaluate accurately. In any case, a relative pain relief of ≥50% seems sufficiently quantifiable to be considered a significant and relevant reduction in pain.

We made an intentional decision to evaluate the immediate pain relief, achieved by the local anesthetic in order to assess the diagnostic value of the injection. The long-term effect of joint injections was not subject of this study, as there are other studies, which were performed regarding this aspect.^28,29^

Major limitations of this study are its retrospective study design and the nonrandomized inclusion of various indications for foot injection and multiple joints. The majority of indications for injections were degenerative in nature: arthritic inflammatory state, osteochondral lesions, and subchondral fracture were rarely or not at all among the indications for injection (Table 1). This can be seen as a selection bias; on the other hand, this can also be seen as an advantage, that independent of the underlying pathology, BME is an expression or a reaction to stress or inflammation within the bone to different kinds of underlying pathologies, which can be used to diagnose the pain-generating joint.^31,32^

As an additional limitation, no sample size calculation was performed and there might have been a selection bias. Our orthopaedic surgeons referred the patients for each joint injection, based on clinical presentation, radiographs, and MRI scan. The injection site was not selected based on MRI findings alone, and our referring physicians were not aware of BME possibly affecting the outcome. However, this could also be interpreted as an advantage of the study. The absence of a control group may limit the ability to assess the contribution of the placebo effect to the efficacy of the join injection. However, administering injections without medication in a control group would expose participants to both radiation and a risk of infection, which, although minimal, was omitted in this study. Additionally, a large number of joints in this study did not show subchondral BME-like signal around the injected joint and can therefore be considered as control joints (n = 46 in reader 1 and n = 50 in reader 2).

Despite its limitations, our study provides valuable insights, highlighting that the extent of subchondral BME-like signal on MRI can predict the diagnostic potential of an intraarticular joint injection, with excellent interrater reliability.

## Conclusion

Pain reduction after intraarticular foot injection correlates significantly with severity of subchondral BME on MRI with excellent interrater reliability and thus can aid clinical decision making.

## Supplemental Material

sj-pdf-1-fai-10.1177_10711007251334097 – Supplemental material for Severity of Bone Marrow Edema on MRI Predicts the Diagnostic Potential of Foot Joint Injections Supplemental material, sj-pdf-1-fai-10.1177_10711007251334097 for Severity of Bone Marrow Edema on MRI Predicts the Diagnostic Potential of Foot Joint Injections by Matthäus Cieciera, Reto Sutter, Stephan H. Wirth, Tobias Götschi and Nadja A. Farshad-Amacker in Foot & Ankle International
